# Feasibility and effectiveness of the implementation of a primary prevention programme for type 2 diabetes in routine primary care practice: a phase IV cluster randomised clinical trial

**DOI:** 10.1186/1471-2296-13-109

**Published:** 2012-11-16

**Authors:** Alvaro Sanchez, Carmen Silvestre, Regina Sauto, Catalina Martínez, Gonzalo Grandes

**Affiliations:** 1 Primary Care Research Unit – Bizkaia, Basque Health Service, Osakidetza, Spain; 2 Quality Unit, Gipuzkoa Health Region, Osakidetza, Spain; 3 The Basque Institute for Healthcare Innovation (O+berri), Osakidetza, Spain

**Keywords:** Pre-diabetes, Primary health care, Prevention, Clinical trial

## Abstract

**Background:**

The objective of this study is to perform an independent evaluation of the feasibility and effectiveness of an educational programme for the primary prevention of type 2 diabetes (DM2) in high risk populations in primary care settings, implanted within the Basque Health Service - Osakidetza.

**Methods/design:**

This is a prospective phase IV cluster clinical trial conducted under routine conditions in 14 primary health care centres of Osakidetza, randomly assigned to an intervention or control group. We will recruit a total sample of 1089 individuals, aged between 45 and 70 years old, without diabetes but at high risk of developing the condition (Finnish Diabetes Risk Score, FINDRISC ≥ 14) and follow them up for 2 years. Primary health care nursing teams of the intervention centres will implement DE-PLAN, a structured educational intervention program focused on changing healthy lifestyles (diet and physical activity); while the patients in the control centres will receive the usual care for the prevention and treatment of DM2 currently provided in Osakidetza. The effectiveness attributable to the programme will be assessed by comparing the changes observed in patients exposed to the intervention and those in the control group, with respect to the risk of developing DM2 and lifestyle habits. In terms of feasibility, we will assess indicators of population coverage and programme implementation.

**Discussion:**

The aim of this study is to provide the scientific basis for disseminate the programme to the remaining primary health centres in Osakidetza, as a novel way of addressing prevention of DM2. The study design will enable us to gather information on the effectiveness of the intervention as well as the feasibility of implementing it in routine practice.

**Trial registration:**

ClinicalTrials.gov NCT01365013

## Background

Type-2 diabetes (DM2) has become one of the main causes of morbidity and early mortality in most countries, associated especially with a higher risk of cardiovascular diseases. Further, it is expected that the prevalence of DM2 will double by 2030 and that this condition will become the seventh cause of death worldwide [1]. Cardiovascular morbidity and mortality in individuals with DM2 is much higher than in the general population. The mortality rate associated with diabetes ranges between 13 and 30 deaths per 100,000 people per year, the main cause of death being coronary disease [2]. It has also been estimated that half of the population of Europe will have hyperglycaemia or diabetes during their lives [3], these conditions becoming more prevalent among both elderly and young people, with a sudden increase in those below 30 years of age [4]. This dramatic increase in the recently diagnosed cases of DM2 and its complications have become an important public health problem and affect almost all populations in both developed and developing countries [1,5].

In recent years, several clinical trials have demonstrated that is possible to prevent DM2 through educational interventions to change lifestyles. Specifically, in high risk subjects, the risk of developing DM2 can be reduced by around 60% after 3 years of these types of intervention [6-8]. Additionally, the effects of interventions focused on changing lifestyles seem to last in the long-term [9,10]. The findings of these studies are revealing. However, the evidence has been obtained from large controlled clinical trials, with well-developed infrastructure and considerable resources. It is not clear whether more realistic interventions, feasible under normal working conditions in health systems, would lead to results similar to those in the aforementioned large trials [11,12]. Therefore, the key question is: can the efficacy demonstrated in clinical trials be replicated in normal clinical working conditions, with much lower resources for the implementation of these types of programmes and interventions?

The step after testing the efficacy of a new experimental intervention, with phase III clinical trials, is the assessment of its effectiveness, efficiency and long-term sustainability under real conditions, with phase IV trials [13]. Unfortunately this step is not at all easy. The widespread adoption of this type of health intervention does not automatically follow from research proving its efficacy, let alone in the specific context of primary health care, characterised by work overload, with shortages of time and training. In recent years, there have been several initiatives assessing the transfer of interventions related to the promotion of healthy lifestyles for the prevention of DM2 to the real context of primary healthcare [14-20]. Some of these have indicated that it is feasible to reduce the risk of developing DM2 in several routine clinical settings. In most cases, however, the studies were not based on group comparisons, were not set up and implemented under real-world conditions in primary care settings or relied in additional resources, and/or did not include the reduction in the incidence of DM2 among the outcome variables. Despite evidence from experimental trials, the translation of lifestyle interventions into routine practice appears to have no effect on diabetes risk reduction [21].

Recently, *The Diabetes in Europe-Prevention using Lifestyle, Physical Activity and Nutritional Intervention* project (DE-PLAN), has been launched within the Spanish National Health Service [22]. This is a public health study focused on evaluating the implementation of a programme developed and implemented in the primary care setting involving: 1) a screening programme for identifying individuals at high risk of developing DM2; 2) an integrated programme with various different intensive interventions for changing lifestyles among high risk individuals; 3) an ongoing intervention programme for maintaining the motivation of participants; and 4) a programme for assessing the feasibility and cost-effectiveness of the aforementioned screening and intervention programmes, in different European primary care systems. The only results published so far concerning the DE-PLAN study conducted in Madrid are exclusively related to the participants in the intervention group, with no comparison group, so that the results obtained cannot be attributed to the programme [23]. One year after the intervention, about 7% of patients in the intervention group developed clinical features of DM2, similar to the figures reported for the control groups in two large DM2 prevention trials [6,7]. More recently, the researchers of the DE-PLAN project in Catalonia [24] have demonstrated that an intensive intervention for the promotion of healthy lifestyles is feasible in the context of primary care and does significantly reduce the prevalence of DM2 among high risk individuals. After a mean follow-up of 4.2 years, the incidence of DM2 was 7.2 and 4.6 cases per 100 people per year, for usual care and intensive intervention groups, respectively (equivalent to a 36.5% reduction in relative risk). In this study, patients were randomly selected previously to inclusion and centres were asked to assign subjects who agreed to take part in the programme consecutively to comparison groups if feasible. The fact that it is an observational study limits its ability to confirm the effectiveness of the programme in real-world primary care settings.

In short, the methodological limitations and mixed results of studies evaluating the effects of primary DM2 prevention programmes in primary care raise doubts as to whether the implementation of these programmes in normal working conditions in health centres would achieve the results observed in experimental trials. Accordingly, prior to widespread implementation, the transfer to real-word settings should be evaluated with a scientifically valid design. The transfer of effective and sustainable interventions to routine clinical practice remains one of the greatest challenges in scientific research in the field of DM2 prevention [12].

The “Prevention of Diabetes in Euskadi” project (PreDE) aims to perform an experimental evaluation of the implementation of a programme for the primary prevention of DM2, through the promotion of healthy lifestyles in patients at high risk of developing the disease, under normal working conditions in primary care. The project is based on the DE-PLAN programme and its methodology, though it has been adapted to the OSAKIDETZA centres. The primary objective is to assess the effectiveness and feasibility of the programme in the routine clinical practice of the centres and health professionals using an experimental a phase IV clinical trial design.

## Methods/design

### Objectives and hypothesis

#### Objectives

The general objective of the study is to perform an independent scientific evaluation of the impact of the implementation of an educational programme for the primary prevention of DM2 in high-risk populations seen in OSAKIDETZA primary care centres. This general objective can be broken down into several specific objectives:

i) to evaluate the results of the intervention programme in terms of reduction in the incidence of DM2, change in dietary habits, and increase in physical activity, among high-risk individuals attending OSAKIDETZA primary care centres in which the programme has been implemented.

ii) to estimate the effectiveness attributable to the intervention, comparing its results with those of patients seen in control centres where patients will receive the current usual care.

iii) to assess the feasibility of the intervention (in terms of coverage, adoption and implementation) in the OSAKIDETZA primary care centres.

#### Hypotheses

i) The implementation of a DM2 prevention programme based on an intervention to promote healthy lifestyles, physical activity and diet, carried out under normal working conditions in the primary care setting over a period of 12 months, will reduce the incidence of DM2 in high risk individuals by 50%, compared to that in a control group of high risk individuals who receive standardised usual care for the prevention of DM2. The expected reduction in the incidence, 50%, a clinically significant difference, is based on previous studies on DM2 prevention in other countries [6,7,24].

ii) In line with the DE-PLAN data from Spain [23], we expect that 50% of the patients exposed to the intervention programme will meet the recommended levels of physical activity (30 minutes of physical activity at least 5 times a week) and consumption of fruit and vegetables (at least 5 portions a day). In the control group, we expect that 25% of patients will meet the minimum recommendations.

### Study design

This is a phase IV randomized cluster clinical trial conducted in 14 Osakidetza primary care centres, randomly assigned to the intervention or control groups. The same system for identifying high-risk patients will be set up in all the centres, based on the administration of the 8-item Spanish version of the validated European Finnish Diabetes Risk Score (FINDRISC) [25]. Non-diabetic patients aged between 45 and 70 years old who are identified as at high-risk of developing DM2 (FINDRISC score ≥14) will be invited to participate in a follow-up programme lasting 24 months with annual medical check-ups. Additionally, a sub-sample of included patients will complete an independent, telephone interview carried out by blind external interviewers on dietary habits and level of physical activity at baseline and again after 12 months.

**Figure 1:**
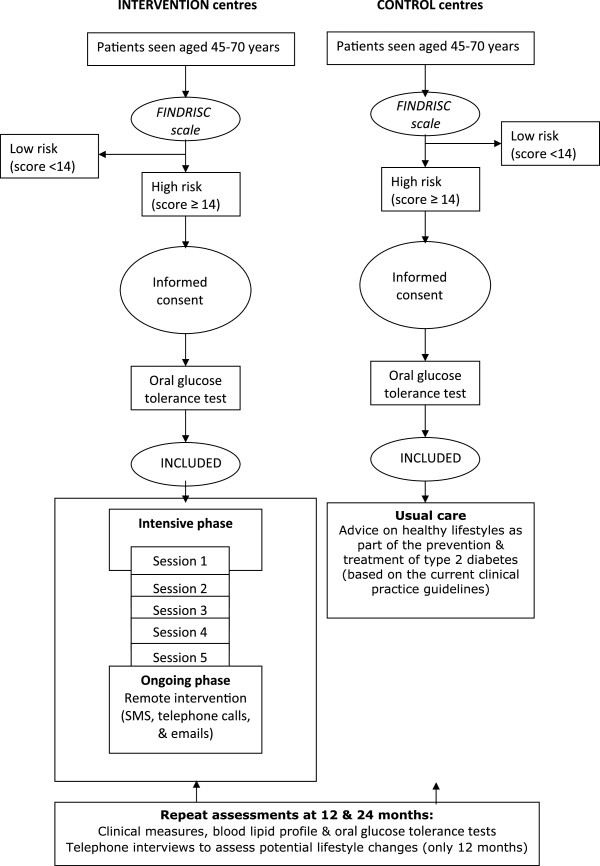
Flow of the study.

Primary care nursing professionals from the seven intervention centres have set up the DE-PLAN programme (for the intervention groups, IGs), adapted to the setting and normal working conditions in the health centres in OSAKIDETZA. Patients found to be at high risk of developing DM2 (FINDRISC score ≥ 14) who then complete the baseline assessments at these intervention centres will be invited to participate in the DE-PLAN programme; while those in the control health centres, will receive usual care for the prevention and treatment of DM2 (Figure 1).

#### Setting

The PreDE initiative is one of the strategic projects of the “Strategy for Tackling the Challenge of Chronicity in the Basque Country” of the Department of Health of the Government of the Basque Country [26]. This Department decided to commission a pilot study of the implementation of the DE-PLAN programme in 14 primary care centres of OSAKIDETZA and fund an independent assessment of the results, to provide a basis for its future dissemination to the remaining primary care health centres, as a new approach to the prevention of DM2 in the Basque Country. This protocol concerns to the design of the independent scientific assessment of the implementation of this prevention programme for diabetes under normal clinical conditions in the OSAKIDETZA primary care centres. The research protocol has been approved by the Basque Country Clinical Research Ethics Committee (Ref. no.: 10/2010). The Basque Healthcare Service (Osakidetza) provides universal free coverage, aside from co-payment for drugs, funded through regional general taxation. In Spain healthcare is a constitutionally-guaranteed right, the public system dominates the healthcare market and primary care services are almost exclusively supplied in public facilities. Each citizen is included in the list of one family physician or pediatrician who offers comprehensive primary care and constitutes the access gate by referral to hospital services. Primary care professionals work in full-time primary health care teams, including family physicians, pediatricians, nurses, and administrative staff hosted in centers and serving with extensive accessibility to a defined geographical area. Healthcare professionals have a civil-servant type labor status and they are paid a fixed salary with a small capitation supplement for physicians.

#### Participants

##### Primary care centres

In each of the seven health regions in which the Basque Health Service is organized, we selected a convenience sample of two centres, similar in terms of structure and population covered. After explaining the objectives of the project and working plan in a presentation session in each of the candidate centers, those professionals who wanted to collaborate signed a written commitment form individually. The 14 centres have been randomly assigned to the intervention and control groups, stratified on a 1:1 basis for each health region, using computer-generated random numbers provided by the Primary Care Research Unit of Bizkaia, such that in each health region there is one intervention and one control group.

##### Primary healthcare users

All users aged between 45 and 70 years old, without diabetes, who attend one of the participating health centres for any reason case during the period of implementation of the programme are eligible to participate. Exclusion criteria are: Previous diagnosis of diabetes mellitus other than gestational; To participate regularly in vigorous exercise programs; Having a chronic disease that makes survival at 6 years unlikely; Patients with diseases that could interfere with the metabolism of glucose.

#### Recruitment process

The 14 centres will implement a system for identification and screening of all eligible patients based on the FINDRISC scale. Eligible individuals with no exclusion criteria who have a score ≥ 14 on the FINDRISC scale, indicating that they are at high risk of developing DM2, will be invited to participate in the study and given the patient information sheet. This explains that they will be followed-up for a period of 24 months with annual medical check-ups consisting of blood lipid profile and glucose tolerance tests. In addition, to those belonging to intervention centres it provides information on the intervention protocol. Patients who agree to participate and sign an informed consent form, will be invited to perform an oral glucose tolerance test (OGTT) with 75 g of glucose in accordance with the WHO guidelines and a determination of lipid profile and HbA1C. Centre’s nurses will measure body weight, height, and waist circumference. Patients who after the test are diagnosed with DM2 will be seen by their family physician and excluded from the study. High-risk patients with glucose levels below 200 mg/ml in the OGTT will be included in the study.

A sub-sample of consecutive included patients once a full implementation of the program in centres is guaranteed (e.g., three months after study initiation), will undergo an independent baseline assessment by blind certified interviewers of their dietary habits and level of physical activity. In order to minimise selection bias, interviewers will make up to six telephone calls at different times of day and on different days in the week in an attempt to contact these individuals, until they are interviewed or are classified as “not contactable”. Non-contactable patients will not be substituted.

#### Intervention standardisation

##### Common treatment for both groups in the study

Both comparison groups will implement the same procedures for screening and initial assessment (clinical examination, blood lipid analysis, and an OGTT with 75 g of glucose, following the WHO guidelines) as well as annual check-ups at the health centres.

##### DE-PLAN educational intervention group

The DE-PLAN educational intervention programme for promoting healthy lifestyles (mainly diet and physical exercise) is carried out by nurses. The intervention is designed to achieve at least three out of the five following objectives: 1) maintenance of ideal body weight in people with normal weight and loss of >5% of body weight in those who are overweight or obese; 2) fat intake <30% of the daily energy intake, 3) intake of saturated fats <10% of the daily energy intake, 4) intake of fibre >15 g/1000 kcal/day, and 5) physical activity >4 hours per week. The intervention is performed in two phases:

a) Intensive intervention through educational sessions in small groups on the modification of unhealthy and adoption of healthy habits. This phase comprises four group sessions of 1.5 hours each. Its objective is to motivate participants to adopt healthy lifestyle habits and provide information concerning the most suitable diet and exercise, as well as agreeing on specific objectives for eating habits and physical activity.

b) Continuous reinforcement for maintaining motivation through regular contact with participants. Once the intensive education intervention programme has been completed, the participants will regularly (at least once every 6 weeks) receive reinforcing educational information via telephone calls, e-mails, etc. from nurses through a health communication platform.

The nurses in charge of the intervention group have received a 14-hour training course focused on the content and procedures for the educational intervention. Additionally, they have received a five-hour training course on the procedures for screening and identifying patients.

##### Control group

The nurses in charge of the control group will continue the current practice and provide the usual care with advice on healthy habits as part of the prevention and treatment of DM2, based on the current clinical practice guidelines of Osakidetza. Given that the system for screening and identifying patients is the same in both groups, the healthcare professionals of the control groups also received the five-hour training course.

#### Evaluation of results

##### Effectiveness of the intervention among patients

The primary outcome variable will be the relative risk of developing DM2, comparing the accumulated incidence (assessed by the OGTT) in the intervention and control groups after 24 months. The secondary outcome variables will be observed changes at 12 months in a sub-sample of intervention and control patients in: the proportion of patients who meet the recommended aerobic physical activity levels (moderate-intensity physical activity for ≥ 30 minutes 5 days/week or vigorous intensity activity for ≥ 20 minutes 3 days/week), derived from the 7-day Physical Activity Recall questionnaire [27]; and the proportion of patients who consume ≥ 5 servings of fruits and vegetables, assessed by the Mediterranean Diet Adherence Screener (MEDAS) used in the ‘*Prevención con Dieta Mediterránea* (PREDIMED)’ study [28]. Lastly, potential effect-modifying variables will be also measured: age, sex, and level of education, among others.

##### Feasibility of the implementation of the programme and its components

The following indicators will be assessed: a) adoption of the programme, in terms of percentage of health professionals that carry out various different components of the intervention; representativeness, dropout rate, causes and degree of influence of the dropout rate on outcomes; b) reach of the programme, in term of percentage of patients exposed to the programme, dropout rate, causes and degree of influence of the dropout rate on the outcomes; and c) implementation of the components of the programme, in terms of degree to which participating patients had received the various different components of the programme as planned.

#### Data quality and management

The coordination, control of quality of processes related to the study, data management and quality assurance will be the responsibility of the Primary Care Research Unit of Bizkaia. The OGTT will be performed in the corresponding health centres or reference laboratory, and results recorded in patient medical records. Changes in lifestyle habits of participants will be assessed by trained professional interviewers, blind to the assignment of patients to the comparison groups, under the supervision of the research team. Data will remain anonymous and only be used for the purpose of this research. Confidentiality of the subjects participating in the study will be maintained in compliance with the Spanish Organic Law 15/1999 on Personal Data Protection (of the 13^th^ December).

#### Sample size

Considering the most conservative results found among high-risk patients in DM2 prevention projects in other countries [6,7] and the most recent data reported from the DE-PLAN project in Catalonia [24], the expected two-year incidence is 15% in the control group. Assuming that the percentage of people diagnosed with DM2 at 24 months will be 15% and 7.5% in the control and experimental groups respectively, and assuming a proportion of 60% recruited patients in the control group with respect to the total sample, to achieve a power of 80% to detect a 50% reduction in the incidence of DM2 as significant, by a Chi square test for independent samples with a two-sided p < 0.05, we need a recruitment under routine conditions of 355 and 237 patients in the control and intervention centres, respectively. However, if we assume that 15% of participants were to be lost to follow-up and an intra-class correlation of 0.01, given that this is a cluster-randomised clinical trial, the number of patients required would be 653 in the control group and 436 in the intervention group, that is, 1089 in total. A sample of 360 patients, 180 in each comparison group, will provide a statistical power of at least 90% to detect, as significant with p <0.05, differences of 50% in the percentage of patients who follow the minimum recommendations of physical activity and servings of fruit and vegetables.

#### Analysis

Statistical analysis will be carried out using the SAS package (version 9.2; SAS Institute, Cary, NC; 2008). The percentages of diagnosed diabetes at 12 and 24 months after the inclusion in the study will be calculated and the level of association with exposure to the educational intervention, considering the potential confounders, will be assessed. Multiple logistic regression analyses will be performed on the percentage of patients diagnosed of diabetes at 24 months, adjusting for intervention group, socio-demographics (age, sex, etc.), and baseline clinical variables (BMI, BP, lipid profile values, etc.). Odds ratios (ORs) and the corresponding 95% confidence intervals (CIs) will be calculated.

Survival analysis will be used to compare the time until diagnosis of diabetes in the different groups. To assess the univariate association of the time until diagnosis of diabetes with the comparison groups and with other possible predictors, cumulative survival probabilities in the 24 months will be estimated and compared using Kaplan-Meier curves along with the log-rank and Wilcoxon tests (SAS PROC LIFETEST, version 9.2, SAS Institute; 2008). Person-years will be the sum of time under follow-up for all participants before diabetes diagnosis or end of follow-up if free of developing diabetes. Subjects who withdrew from the study will be considered to be at risk for diabetes until their last oral glucose tolerance. However, an effort will be made in order to ascertain a diabetes diagnosis at the end of the study period reviewing patients’ charts. Adjusted hazard ratios (AdHRs) and 95% CIs will be estimated to compare diagnosis of diabetes risks between patients in different groups using Cox proportional hazards models introducing all the variables studied in the bivariate analysis as potential confounders. The proportional hazard assumption will be assessed using graphical techniques and a statistical test based on Schoenfeld partial residuals of the model. In the case of covariates for which the proportional hazard assumption is not satisfied, the Cox model will be extended with time-dependent variables allowing hazard ratios to change over time. Finally, to model the clustered structure of data, a common random effect will be introduced into the Cox’s proportional hazards model. This estimation will be carried out by adaptive Gaussian quadrature method implemented in SAS PROC NLMIXED.

As for the secondary outcome variables, the differences between the groups will be estimated and 95% confidence intervals calculated. The mean changes of the continuous variables will be compared by Student’s t-tests, while differences in proportions will be compared by chi Square tests. In the case of non-normal distribution of data non-parametric tests will be used. Generalized mixed-effects models will be used to estimate baseline and multivariate adjusted between-group differences, adjusted odds ratios (AORs), and 95% confidence intervals (CIs) at the patient level, taking into account the hierarchical and multi-center structure of data.

## Discussion

This study is to be an independent assessment of the results of the implementation of a prevention programme for DM2 in high-risk populations seen in 14 primary care centres in the Basque Health Service, Osakidetza, commissioned by the Department of Health of the Government of the Basque Country. The scientific evidence provided by this study may be unique internationally as it has not, so far, been demonstrated that a programme for preventing DM2, implemented under real conditions in primary health care centres, is able to prevent the development of DM2 in high-risk patients.

Despite the promising results of the studies of DM2 prevention in routine clinical setting available to date [14-18,21], transferring effective, sustainable and acceptable interventions to routine clinical practice in primary care remains a challenge. The transfer and application of research to clinical practice is a complex process [29] that requires planning at individual and organisational levels [30]. On the other hand, health professionals work in specific organisational and structural contexts within a system and there are a wide range of factors that may facilitate or impede change [31-33].

Despite the Basque Health System having already entered into a period of change due to new health policies focused on addressing the issues associated with chronicity [26], the implementation of any new programme in routine clinical conditions is an extra challenge that requires those involved to adapt and barriers to change to be overcome [32,33]. The strategy for the implementation of the present programme for preventing diabetes in routine practice has mainly involved the training of nurses. Additionally, materials and resources for data management have been provided and health professionals have been encouraged to optimise the way they work together. It remains to be seen whether these passive strategies are enough to enable the successful implementation of the programme, and ensure that it is sustainable over time by the health centres, has sufficient reach in the target population, and is effective in terms of patient health. Specifically, in the light of results of other studies, various difficulties can be envisaged that would influence the implementation of the programme and potentially its results: these relate to the degree of adoption among primary care health professionals, patient recruitment during routine clinical practice in the intervention centres, including a lack of willness of patients to participate, problems in the organisation of educational groups, and losses to follow-up, as well as the many external barriers to change with regards to lifestyle habits [12,21].

The present study is intended to generate valid scientific data concerning the effectiveness of a programme promoting healthy lifestyles for the prevention of type 2 diabetes in the real world of primary care. It also sets out to assess the feasibility of the programme in terms of reach, adoption and implementation in the routine clinical context and provide data on the many strategies used in the course of its implementation. The results obtained, if positive, will be used to facilitate the process of rolling out the programme to all the primary care centres of the Osakidetza as a new approach to preventing DM2.

## Competing interests

The authors declare that they have no competing interests.

## Authors’ contributions

GG and AS conceived the idea and are the study guarantors. They are primarily responsible for the study design and planning, obtained funding, and will be responsible for project coordination and supervision, analysis and interpretation of results and manuscript preparation. CS and RS collaborated in the study design, obtained funding, and will be responsible for study coordination, interpretation of results and manuscript preparation. AS, GG and CS will be responsible for the analysis of results and critically reviewed the manuscript. All contributors approved this version submitted for publication to ***BMC Public Health.*** All authors read and approved the final manuscript.

## Pre-publication history

The pre-publication history for this paper can be accessed here:

http://www.biomedcentral.com/1471-2296/13/109/prepub
